# Synergy between Nitrogen Removal and Fermentation Bacteria Ensured Efficient Nitrogen Removal of a Mainstream Anammox System at Low Temperatures

**DOI:** 10.3390/toxics12090629

**Published:** 2024-08-26

**Authors:** Jiaru Zhi, Guocheng Ma, Xueqing Shi, Guoqing Dong, Deshuang Yu, Jianhua Zhang, Yu Zhang, Jiawen Li, Xinchao Zhao, Haizheng Xia, Xinyu Chen, Zhuoya Tian, Yuanyuan Miao

**Affiliations:** 1 School of Environmental Science and Engineering, Qingdao University, Qingdao 266071, China; 18247474063@163.com (J.Z.); 15650171526@163.com (G.M.); donggq0228@163.com (G.D.); qdydsh@163.com (D.Y.); zhang18763442329@163.com (Y.Z.); 17806275827@163.com (J.L.); 13196243265@163.com (X.Z.); 2 School of Environmental and Municipal Engineering, Qingdao University of Technology, Qingdao 266520, China; shixq85@163.com (X.S.); zhangjianhua@qut.edu.cn (J.Z.); 15133221368@163.com (H.X.); 18774960829@163.com (X.C.); miaoyy1993@163.com (Z.T.)

**Keywords:** mainstream anammox, low temperature, fermentation, nitrogen removal, microbial correlation networks

## Abstract

Simultaneous partial nitrification, anammox, denitrification, and fermentation (SNADF) is a novel process achieving simultaneous advanced sludge reduction and nitrogen removal. The influence of low temperatures on the SNADF reactor was explored to facilitate the application of mainstream anammox. When temperature decreased from 32 to 16 °C, efficient nitrogen removal was achieved, with a nitrogen removal efficiency of 81.9–94.9%. Microbial community structure analysis indicated that the abundance of *Candidatus Brocadia* (dominant anaerobic ammonia oxidizing bacteria (AnAOB) in the system) increased from 0.03% to 0.18%. The abundances of *Nitrospira* and *Nitrosomonas* increased from 1.6% and 0.16% to 2.5% and 1.63%, respectively, resulting in an increase in the ammonia-oxidizing bacteria (AOB) to nitrite-oxidizing bacteria (NOB) abundance ratio from 0.1 to 0.64. This ensured sufficient nitrite for AnAOB, promoting nitrogen removal. In addition, *Candidatus* Competibacter, which plays a role in partial denitrification, was the dominant denitrification bacteria (DNB) and provided more nitrite for AnAOB, facilitating AnAOB enrichment. Based on the findings from microbial correlation network analysis, *Nitrosomonas* (AOB), *Thauera*, and *Haliangium* (DNB), and *A4b* and *Saprospiraceae* (fermentation bacteria), were center nodes in the networks and therefore essential for the stability of the SNADF system. Moreover, fermentation bacteria, DNB, and AOB had close connections in substrate cooperation and resistance to adverse environments; therefore, they also played important roles in maintaining stable nitrogen removal at low temperatures. This study provided new suggestions for mainstream anammox application.

## 1. Introduction

In the anammox process, ammonium and nitrite undergo a conversion into N_2_ along with a limited production of nitrate under anoxic conditions [1,2]. Compared to traditional nitrogen removal techniques, anammox requires no external carbon source, needs no aeration, and has low sludge production [3,4]. However, the effluent still contains nitrate. To further enhance the nitrogen removal efficiency of the anammox system, the simultaneous partial nitrification, anammox, and denitrification (SNAD) process has been developed, in which denitrification bacteria (DNB) can reduce the nitrate produced by nitrite-oxidizing bacteria (NOB) and anaerobic ammonia oxidizing bacteria (AnAOB). Nevertheless, due to the necessity of introducing carbon sources to facilitate denitrification, the sludge yield is high. Ma et al. (2023) [5] illustrated that the integration of SNAD and fermentation (SNADF) can reduce sludge production and energy consumption, with a nitrogen removal efficiency of 92.8%. Therefore, SNADF was considered a low-cost and efficient sewage treatment process.

Low temperature significantly limits the widespread adoption of the anammox process. During winter, the temperature of wastewater in most regions typically ranges from 20 to 10 °C. However, previous studies have demonstrated that a temperature range of 28 to 32 °C is optimal for the growth of AnAOB [6,7]. The temperature of around 30 °C is optimal for DNB [8]. Laureni et al. [9] discovered that the nitrogen removal decreased significantly by 60% and the maximum anammox activity decreased from 300–600 to 138 ± 38 mg N·L^−1^·d^−1^ with the decrease in temperature from 29 to 15 °C. Furthermore, at low temperatures, NOB are more prone to accumulation, which further deteriorates the nitrogen removal in the system. Therefore, the main challenge in promoting the application of SNADF is to sustain stable nitrogen removal performance in low-temperature environments.

According to previous studies, low temperatures enhanced the efficiency of partial denitrification; although the nitrogen removal efficiency decreased slightly, the accumulation of nitrite during partial denitrification increased the nitrogen removal contribution of AnAOB [10]. Furthermore, Liu et al. [11] successfully performed anammox under low-temperature conditions; with a minimum total nitrogen removal efficiency of 78.2% at 14.1 °C, fermentation bacteria (FB) played a promoting role in facilitating denitrification at low temperatures. Therefore, it was speculated that SNADF had the potential to achieve efficient nitrogen removal at low temperatures, but empirical studies are lacking.

The purpose of this study is to explore the nitrogen removal performance and shifts in the microbial community within SNADF systems with a gradually decreasing treatment temperature. Furthermore, the synergistic relationships among functional microorganisms during temperature reduction are determined through microbial co-occurrence network analysis.

## 2. Materials and Methods

### 2.1. Reactor Configuration

A laboratory-scale sequencing batch reactor (SBR, working volume: 6 L) was used in the experiment. The SBR had a volume exchange ratio of 50%. The operational cycle lasts for 6 h, consisting of feeding (5 min), anoxic mixing (40 min), intermittent aeration (270 min), and settling and drainage (45 min). The reactor temperature was controlled by a heating rod. The intermittent aeration was regulated by a time relay, with aeration occurring for 9 min followed by a non-aeration period of 21 min. The dissolved oxygen (DO) level during the aerated stages was maintained in the range of 0.4–0.6 mg/L with a compressor and rotameter. The experiment comprised five phases with different temperatures: phase A (1–20 d, 32 °C), phase B (21–40 d, 28 °C), phase C (41–60 d, 24 °C), phase D (61–80 d, 20 °C), and phase E (81–100 d, 16 °C).

### 2.2. Seeding Sludge and Wastewater

The seed sludge was taken from a SNADF reactor [5] and operated in an intermittent aeration with a running pattern identical to the reactor in this study. The synthetic wastewater contained NH_4_^+^-N (59.2 ± 3.5 mg N/L) and COD sodium acetate (182.3 ± 32.1 mg/L) with a pH of 7.7–7.9 and a temperature of 32 °C. The nitrogen loading rates ranged from 111.4 to 125.4 g N/(m^3^·d), and the organic loading rate ranged from 428.8 to 300.4 g COD/(m^3^·d). During the stable period, the reactor achieves a TN removal efficiency of 92.8 ± 6%.

In this study, the pH was maintained at 7.5 ± 0.3. The synthetic wastewater containing NH_4_^+^-N (58.6 ± 3.2 mg N/L) and chemical oxygen demand (COD, 178.3 ± 22.7 mg/L) in the form of NH_4_Cl and sodium acetate. The SNADF reactor had a nitrogen organic loading rate ranging from 110.8 to 123.6 g N/(m^3^·d) and organic loading rate ranging from 402 to 311.2 g COD/(m^3^·d). The other additional constituents of the wastewater included the following (mg/L): KHCO_3_ (400), KH_2_PO_4_ (22), MgSO_4_·7H_2_O (300), CaCl_2_·2H_2_O (180), and trace element solutions (1 mL/L) [12]. The SNADF reactor operated under long sludge retention time (SRT), with no sludge discharge in the operation period. The mixed liquor suspended solids (MLSS) remained between 5763 mg/L and 6780 mg/L. The sludge volume index (SVI) of the reactor fluctuated between 128.3 and 137.7 mL/g.

### 2.3. Analytical Methods

Influent and effluent samples were collected daily with 0.45 μm filters. The levels of NH_4_^+^-N, NO_3_^–^-N, and NO_2_^–^-N were measured by standard methods [13]. The COD concentration was determined using a rapid test method (Lian-hua Tech., 5B-3A, Beijing, China). A portable detector (WTW3420, Munich, Germany) was used to determine the DO, pH, and temperature.

### 2.4. Microbial Diversity Analysis

On days 20, 40, 60, 80, and 100, sludge samples were collected from the SNADF system. The quantification of ammonia-oxidizing bacteria (AOB), and of *Nitrobacter* and *Nitrospira* (NOB), in each sludge sample was detected by qPCR [14,15]. Furthermore, the five samples underwent Illumina high-throughput sequencing at Shanghai Majorbio Biopharm Technology Co., Ltd. (Shanghai, China). The raw sequences can be found in the NCBI Sequence Read Archive, accessible via accession number SRP469279.

### 2.5. Statistical Analysis and Network Construction

The richness and diversity of the bacterial community were evaluated by calculating the Chao, Ace, Simpson, and Shannon index values. Using the online MENA, the correlation matrix of 63 functional bacteria was calculated by Pearson’s correlation (r > 0.8, *p* value < 0.05), and the analyses of module separation and module calculation were implemented into three modules (M1, M2, M3). The correlations between key functional microbial communities and temperature were analyzed by the Networkx (version 1.11) software. Visualization analysis was implemented using Cytoscape (version 3.9.1).

## 3. Results and Discussion

### 3.1. Nutrient Removal Performance with Decreasing Temperature

**Figure 1:**
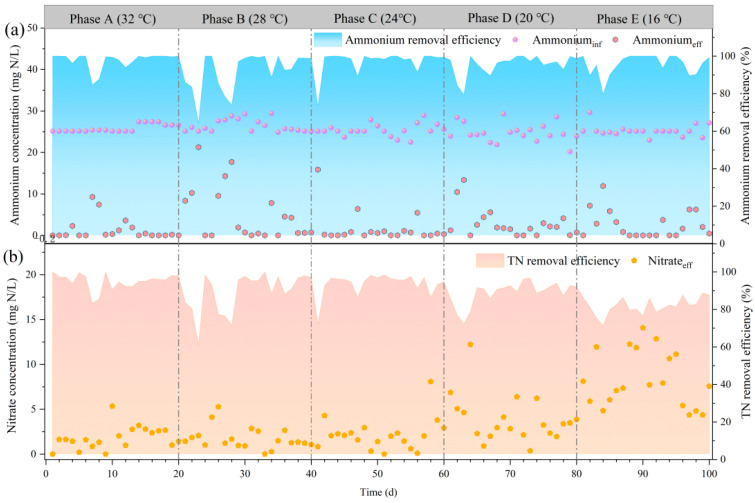
Performance of the SNADF system at decreasing temperatures. (**a**) Influent ammonium, effluent ammonium, and ammonium removal efficiency; (**b**) effluent nitrite and total nitrogen removal efficiency.

During phases A (32 °C), B (28 °C), and C (24 °C), the average ammonium concentrations in the effluent were 1.4, 5.2, and 1.7 mg N/L, respectively (Figure 1a). When the temperature decreased, the ammonium removal efficiency of the reactor decreased for a short period and then recovered rapidly. During phases A–C, there was no significant change in the effluent nitrate concentration; the average effluent nitrate concentrations were 1.8, 1.4, and 2.2 mg N/L, respectively, with TN removal efficiencies of 94.9%, 89.1%, and 93.6%, respectively. This suggested that the decrease in temperature from 32 to 24 °C only had a slight effect on the SNADF system. During phases D (20 °C) and E (16 °C), the ammonium concentrations in effluent were 2.8 and 2.6 mg N/L, respectively, suggesting that the degradation of ammonium was hardly affected by the low temperature. The average effluent nitrate concentration slightly increased to 3.9 and 8.3 mg N/L, with a TN removal efficiency of 88.9% and 81.9%, respectively. The increase in nitrate in the effluent might be due to the enrichment of NOB under low temperature. The reactor’s effluent COD concentration maintained at 22 ± 6.5 mg/L. Based on these findings, under a low temperature (16 °C), the SNADF process could maintain efficient nitrogen removal performance.

Under low-temperature (16 °C) conditions, SNADF would achieve efficient nitrogen removal (81.9%), which showed more stable nitrogen removal than that in previous studies. According to reports, the average nitrogen removal efficiency of the AOA system of AOA was 70.8% at 16 ± 0.4 °C [16]. For IFAS-PN/A reactors, the TN removal efficiency decreased from 79.4 ± 3.0% to 65.9 ± 8.8% when the temperature dropped from 25 °C to 15 °C [17]. In the anammox MBR and UASB reactors, the highest nitrogen removal efficiency was 58.6% and 76.1%, respectively, when the temperature was controlled at 15 °C [18,19]. In MBR-PN/A reactors, the corresponding TN removal decreased to 75.0 ± 7.6% and NH_4_^+^-N removal decreased to 62.9 ± 6.1% with a temperature reduction to 15 °C [20].

### 3.2. Bacterial Community Analysis

#### 3.2.1. Variations in Diversity and Richness of the Bacterial Community

**Figure 2:**
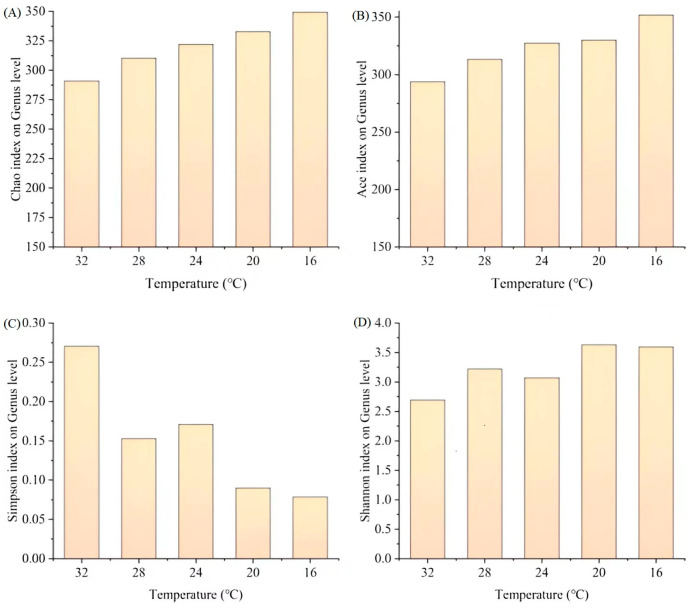
Changes in richness (Chao (**A**) and Ace index (**B**)) and diversity (Simpson (**C**) and Shannon (**D**) indexes) of the microbial community.

To better understand the mechanism underlying the stable nitrogen removal performance of the SPDAF reactor under low temperatures, variations in the microbial community were analyzed. The Ace and Chao indices reflect the bacterial richness in the SNADF system; the larger the index value, the higher the species richness [21]. As temperatures decreased from 32 to 16 °C, the Chao and Ace index values increased from 290.90 and 293.66 to 349.25 and 351.68, respectively (Figure 2A,B). Shannon and Simpson were frequently employed to assess the diversity of the bacterial community [22]. Larger values of the Shannon index indicated a higher level of diversity in the bacterial community, whereas the opposite is true for the Simpson index. During the experimental period, a decrease in Simpson from 0.27 to 0.08 was observed (Figure 2C), while Shannon increased from 2.70 to 3.59 (Figure 2D). These data indicated that the community richness and diversity were higher under low temperatures. A higher diversity of microorganisms is more conducive to resisting environmental interference and promoting system stability [23]. The findings imply that the increase in richness and diversity of the bacterial community might contribute to the stable nitrogen removal efficiency under low temperatures.

#### 3.2.2. Variations in Abundance of Functional Microbial Communities

**Figure 3:**
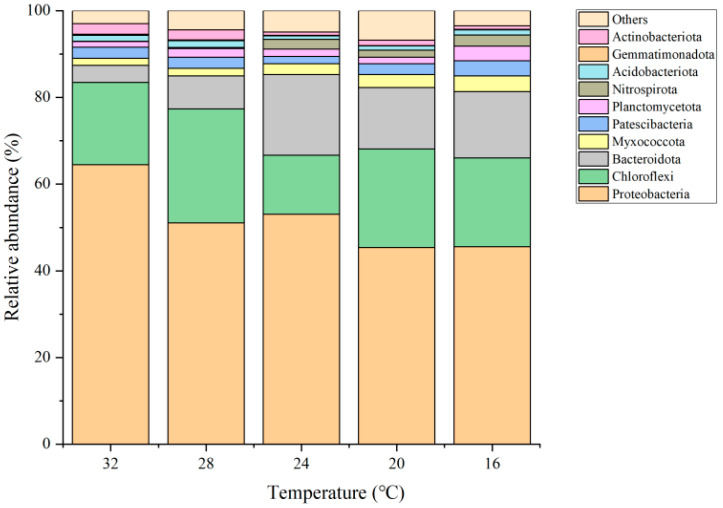
Abundances of microbial phyla.

Bacteroidota, Chloroflexi, and Proteobacteria were the dominant phyla in the SNADF system (Figure 3). When the temperature decreased from 32 to 16 °C, a reduction in the relative abundance of Proteobacteria, which are affiliated with DNB, NOB, and AOB [24], was observed (64.5% → 45.5%). The abundance of Planctomycetes, comprising anammox microorganisms, more than doubled [25], with an increase from 2.3% to 5.2%. The abundances of Chloroflexi and Acidobacteriota, which are associated with heterotrophic denitrification [26], varied from 19% and 1.3% to 22.4% and 1.1%, respectively. The abundance of Bacteroidetes as the main component of hydrolyzed bacteria increased significantly from 3.9% to 15.3% [27]. Overall, the phyla associated with nitrogen removal exhibited high abundances, and a significant increase in phyla related to anammox and hydrolysis was observed.

**Figure 4:**
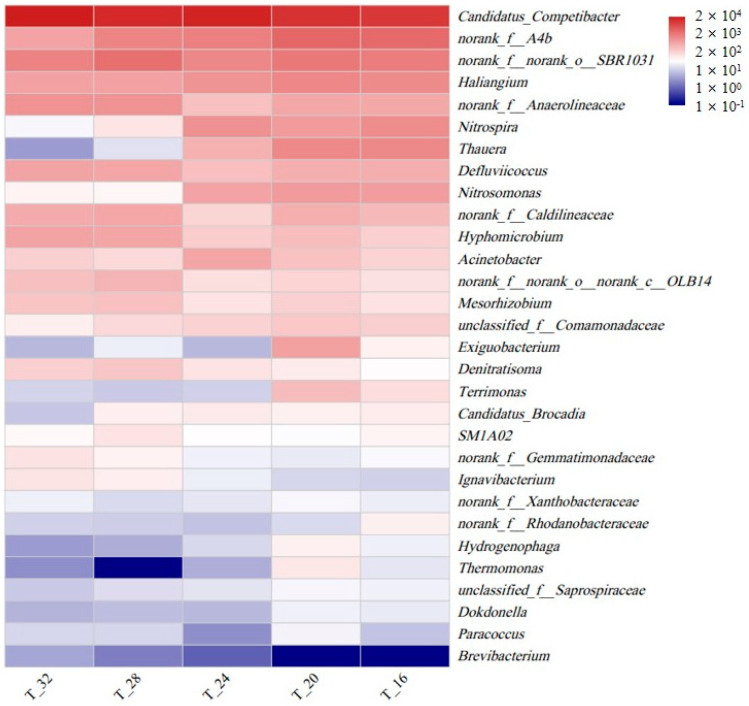
Heat map showing the genera of the reactor.

At the genus level, the distribution of bacterial populations was described in relation to different temperatures (Figure 4). With a decrease in temperature (32 → 16 °C), the abundance of *Candidatus Brocadia* increased from 0.03% to 0.18%, indicating that the abundance of AnAOB was not negatively affected. According to previous studies, *Candidatus Brocadia*, as the main AnAOB, can adapt to low temperatures [22], which could be one of the factors contributing to the stable abundance of AnAOB at low temperatures.

**Figure 5:**
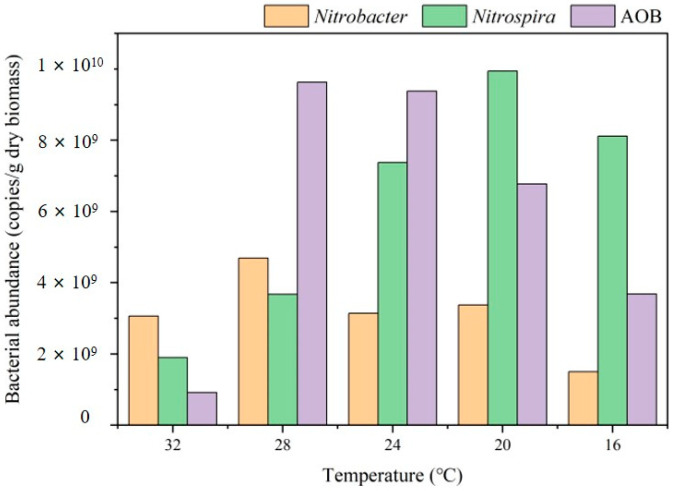
Gene copy numbers of AOB, *Nitrobacter*, and *Nitrospira* through qPCR.

The abundance of *Nitrosomonas*, a dominant AOB, significantly increased from 0.16% to 1.6% [28]. The abundance of *Nitrospira*, which belongs to the group of NOB, increased from 1.6% to 2.5%. The abundance ratios of AOB and NOB were 0.1, 0.6, 1.2, 0.74, and 0.64 at 32, 28, 24, 20, and 16 °C, respectively. Overall, there was an increase in the abundance ratio, suggesting that AOB could better adapt to low temperatures than NOB. This study further investigated the fluctuations in the absolute abundances of AOB and NOB. During the experimental period, the abundances of *Nitrobacter* decreased from 3.1 × 10^9^ to 1.5 × 10^9^ copies/g dry biomass (Figure 5). Upon the decrease in temperature from 32 to 20 °C, both the abundances of *Nitrospira* and AOB significantly increased, and the abundance ratio of AOB and *Nitrospira* increased from 0.04 to 0.91, consistent with the high-throughput results. This further revealed that when the temperature decreased, the system could maintain stable partial nitrification, ensuring nitrite supply for AnAOB. This might also be one of the reasons for the stable abundance of AnAOB and nitrogen removal performance. However, it is worth noting that with the temperature decrease to 16 °C, both AOB and NOB abundances decreased significantly. The long-term stability of the reactor under low temperatures therefore requires further investigations.

According to previous reports, free ammonia (FA) and free nitrous acid (FNA) have impacts on partial nitritation and AnAOB in anammox reactors [29]. In this experiment, there was no accumulation of nitrite during the operational process; therefore, the effect of FNA could be ignored. The highest FA concentrations were calculated based on the maximum NH_4_^+^ concentration and pH of the operation cycle. The FA concentration in phases A (32 °C) to E (16 °C) were 1.26, 0.99, 0.75, 0.56, and 0.42 mg/L, respectively, much lower than the thresholds reported in previous studies: The AnAOB was inhibited at an FA concentration of 13–90 mg/L [30,31,32]. The AOB and NOB were inhibited at an FA concentration of 10–105 mg/L [29,32,33] and 4–16.8 mg/L [34,35,36], respectively. Therefore, the FA concentration had less effect on AnAOB, AOB, and NOB in the SNADF reactor.

*Candidatus* Competibacter, which participates in endogenous denitrification as a glycogen-accumulating organism (GAO) [37], maintained the highest relative abundance (24.2–51.2%), providing a stable substrate (nitrite) for AnAOB. In addition, as the temperature decreased, there was a noticeable increase in the abundance (0.1% → 2.9%) of *Thauera*, which was also recognized as a key contributor to partial denitrification [14,38]. This process could significantly decrease NO_3_^−^ levels in the effluent and ensured a stable supply of NO_2_^−^ to AnAOB, thereby enhancing the overall nitrogen removal performance and stability of SNADF.

The fermentation genera *A4b* (1.5–6.5%), *SBR1031* (3.7–6%), and *Anaerolineaceae* (0.6–2.4%) were among the dominant bacteria in the system. They could provide a substrate for denitrification by decomposing organic matter, thereby promoting denitrification, particularly partial denitrification [39]. The enrichment of DNB and FB also contributed to efficient and stable nitrogen removal in the reactor.

To summarize, when temperature was decreased, the AnAOB (*Candidatus Brocadia*) abundance showed an increase, which was beneficial to nitrogen removal since anammox was the main nitrogen removal pathway in the SNADF system. AOB had a greater growth advantage over NOB, which ensured ammonium removal efficiency and provided a stable supply of substrates to AnAOB. The abundance of partial denitrification bacteria (*Candidatus* Competibacter and *Thauera*) remained high and showed a significant increase. Meanwhile, the abundance of FB remained at a high level, further promoting partial denitrification. This greatly prevented the accumulation of NO_3_^−^ and further ensured stable substrates for AnAOB, thereby ensuring efficient nitrogen removal performance.

#### 3.2.3. Network Analysis

**Figure 6:**
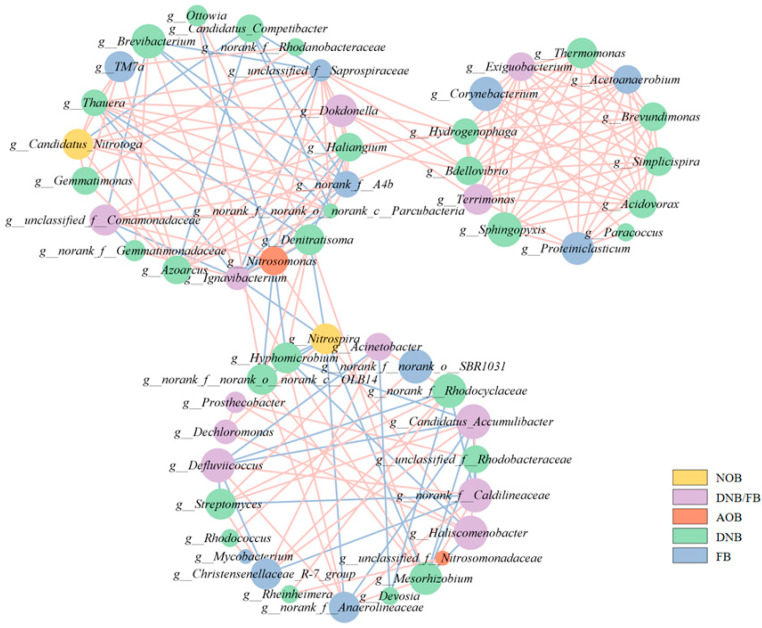
Analysis of the functional microbial correlation network. Positive correlations are indicated by red color, and negative correlations are represented by blue color.

Microbial interactions serve as critical factor in the composition of microbial community [40]. Thus, understanding the interactions of microorganisms is crucial for exploring the mechanism underlying the stable nitrogen removal performance of the SNADF system at low temperatures. Networks were built according to species composition at the genus level (Figure 6). The integrated network consisted of 55 nodes and 209 edges, demonstrating robust (r > 0.8) and statistically significant (*p* value < 0.05) correlations. Other topology indices, the average path distance and average clustering coefficient, were 3.103 and 0.622, respectively. The application of the fast greedy modularity optimization algorithm resulted in the isolation of three distinct modules [41,42], and the modularity was 0.438, indicating that this network exhibited a modular structure [43].

**Figure 7:**
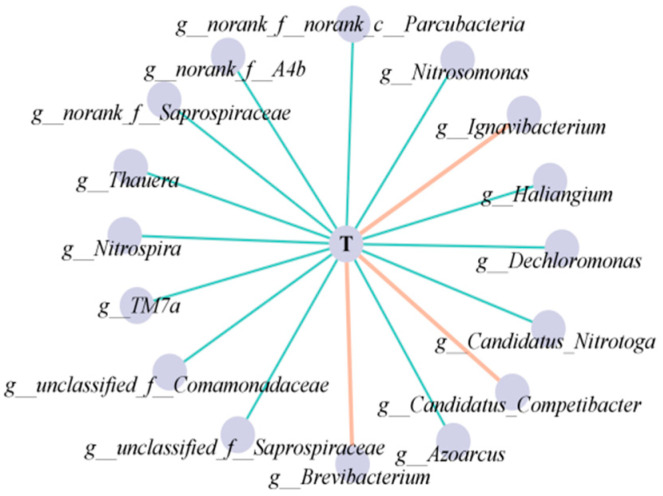
Correlation network between temperature and functional microbes.

The network hubs or keystones, characterized by nodes with degrees exceeding 90% of the maximum degree, were identified as a critical element supporting the sustainability of the community [44]. The keystones were *Nitrosomonas*, *Thauera*, *Haliangium*, *A4b*, and *Saprospiraceae*. Notably, the five keystones exhibited a negative correlation with temperature, indicating that they were enriched under low temperatures, enhancing the stability of the system (Figure 7). This phenomenon constituted one of the significant contributing factors towards achieving efficient nitrogen removal in low-temperature environments. In addition, as the main AOB in the community, the *Nitrosomonas* species—with max betweenness and max stress centrality—were positively correlated with the other three keystones, which might explain the high ammonium removal efficiency at low temperatures. *Thauera* and *Haliangium*, as the genera mainly responsible for partial denitrification, were positively correlated with most FB, whereas *A4b* and *Saprospiraceae*, as FB, were positively correlated with the main DNB such as *Hydrogenophaga* and *Thauera*. This ensured stable partial denitrification at low temperatures, further facilitating stable nitrogen removal at low temperatures.

A module can be seen as a functional unit within a bacterial community, wherein populations might exhibit similarities in their ecological niches [45]. This indicated that the microbial species in M1, M2, and M3 form a network of interactive modules with more efficient and dense material and energy flow. In M2, composed solely of FB and DNB, all populations showed a positive correlation. This suggested highly intimate fermentation–denitrification synergistic interactions between FB and DNB within M2, ensuring the stable nitrogen removal capability of AnAOB. In addition, *Thermomonas* (DNB) having cold resistance can secrete protective EPS, further promoting the interaction between AnAOB and other bacteria [46]. The M1 was mainly composed of DNB and FB. All five keystones appeared in M1 and were positively intercorrelated. The M1 served as a bridge that connected *Haliangium*, *Saprospiraceae*, etc. with M2 and was connected to M3 by *Denitratisoma*, *Nitrosomonas*, etc. These results indicated the synergistic relationship between nitrogen removal bacteria and FB, including substrate supply and resistance to adverse conditions, ensuring the stability of the system at low temperatures.

To summarize, according to biological co-occurrence network analysis, AOB had positive correlations with other key species, and Nitrosomonas (AOB) showed a significant increase during temperature decline. This provided a stable NO_2_^−^ substrate for AnAOB. Additionally, there was a positive correlation between FB and DNB. Dominant DNB (*Candidatus* Competibacter and *Thauera*) were involved in partial denitrification, which also ensured substrate security for AnAOB. FB breaks down macromolecules into micromolecules, supplying carbon sources to DNB [47], and slowly releases carbon sources, promoting synergy between partial denitrification and anammox [33]. Therefore, the overall nitrogen removal performance and stability of SNADF was enhanced.

## 4. Conclusions

With the decrease in temperature from 32 to 16 °C, the SNADF system maintained a stable nitrogen removal efficiency of 81.9%. Microbial community structure analysis indicated that with decreasing temperatures, the abundance of *Candidatus Brocadia* (AnAOB) increased from 0.03% to 0.18%. Although the abundance of NOB increased, AOB had a greater growth advantage over NOB and provided stable nitrite for AnAOB. Partial denitrifying bacteria were the dominant DNB and also provided nitrite for AnAOB, ensuring nitrogen removal. Network analysis indicated a synergistic relationship between DNB and FB, including substrate supply and resistance to adverse conditions, which further enhanced the capability of AnAOB to adapt to adverse environments and ensured the stability of the system at low temperatures.

## Data Availability

Dataset available on request from the authors.
